# The Right Policy. Choose Honest Workers and Trust Them

**Published:** 1918-09-28

**Authors:** 


### A QUESTION OF ATTITUDE.

### The Right Policy. Choose Honest Workers and Trust Them.

It is remarkable with what divergent attitudes different people view the same thing. A German newspaper recently remarked that it was dreadful to contemplate the nation's being governed by a Parliament. President Wilson, on the other hand, has inspired a hundred millions to fight to the death in order to make the world safe for democracy. It is a question of attitude. Some people think that they are born to govern the others, and there are those who resent this and desire to manage their own affairs. I sometimes think that the announcement which publicans make to the world—"licensed to sell beer," etc.—may possibly some day be suitably adapted for other use, and that a number of the population may elect to wear a badge bearing the inscription "licensed to be alive." There are nurses who will gladly adopt the device as a protection from certain of their own order. These things become apparent as the profession becomes articulate.

My remarks are *à propos* of an illuminating letter which appeared in last week's Hospital signed by "A Fully Trained 'London' Hospital Nurse." Read it, if you have not already done so. I have read it more than once. It is brief, but every line displays the writer's attitude, and a wise man would have little difficulty in approximating her general attitude towards many problems with which the world is now occupied, public and domestic. I do not know this lady's name or her place of habitation, but I have arrived at the conclusion that she is a snob. This word I seldom use, because it is so often misused, but it is a good old English word, and has its true and proper application. A snob is a superior person who sub-consciously believes that when all is said and done there is one only person of real importance in the world. The writer suggests that all my information comes from nurses of the domestic servant type. On this I make no comment. My correspondents are quite well able to appreciate the situation. The remark, however, which tickles my fancy most is one which is applied to the whole profession. "If the nurses lived out," she observes, "on the least pretext such as slight headache, or in cold, wet, or snowy weather she [meaning they] would not turn up in the morning." The whole nursing profession, according to this lady, is composed of potential malingerers. Give them "the least pretext," and they will desert the sick and dodge their duty.

I regret to observe that this attitude is not confined to "A Fully Trained 'London' Hospital Nurse," and wherever it is found it indicates unbounded egotism and a corresponding lack of faith in the rest of humanity. One of my earliest recollections is an occasion in my own home where for a special reason a charwoman was asked to come very early to work. She was poor, as charwomen usually are, and she awoke the household on a bright moonlight night at three o'clock. The average "general" would probably regard her as not quite within her social circle, but at all events she was not watching for a pretext.

I know these superior people, and, as the Americans say, I have no use for them. Doubtless they will some day arrive in heaven and there have special apartments provided with large magnifying mirrors. Otherwise it would not be heaven! My theology consists very largely of salvation by faith—faith in my fellow-creatures, whether they be of the domestic servant class or "like the shop-girl" to whom the "Fully Trained 'London' Hospital Nurse" contemptuously alludes, or even those women who devote their lives to nursing the sick. And I think, moreover, that the experience of many matrons and staff sisters is that higher results are obtained by a trustful attitude than by rigid surveillance and the iron heel. Taking it generally, with all its faults, it is a fairly honest world. There are thieves and pickpockets, but there are a great many honest folk, even among nurses.

I should not say so much about the letter of this anonymous correspondent were it not that she represents a type by no means uncommon. The burden of her song is that nurses have no grievance, and if they have one they should hang it up until the war is over. The patriotic stunt is the refuge of a motley crowd. But I challenge the writer or aught else to point to any body of people, men or women, who have done more to win the war than British nurses, the women who "on the least pretext" would avoid their duty.

By the way, it would be interesting to know exactly what are the war aims of this correspondent. It does not necessarily follow that victory will bring with it that measure of consolation which she expects. Is her dream to make the world safe for democracy—for those to whom in her letter she refers, the domestic servant, the shop-girl, miners, and factory hands? I fear lest she may suffer disappointment. The war as I see it is a war of attitude. It is no petty quarrel, but a struggle to the death between one set of people who seek to be able to grant licences, if it so pleases them, to the remainder of the human race to be alive and to breathe the air of heaven, and another set who consider that this round world of ours was made for God's creatures one and all, even if some of them are domestic servants and shop-girls.

Now, having said all this, may I add what may be of some importance? In my remarks of August 31 to which "A Fully Trained 'London' Hospital Nurse" refers, I made no recommendation that nurses should live out. I made no reference to nurses living out. I merely quoted from the letters of ten correspondents, and it was they who ventured to express their views as to their ideals of freedom.

Verily there are Huns in the Homeland.

IERNE.

